# *CYP2C19 *and *ABCB1 *gene polymorphisms are differently distributed according to ethnicity in the Brazilian general population

**DOI:** 10.1186/1471-2350-12-13

**Published:** 2011-01-19

**Authors:** Paulo CJL Santos, Renata AG Soares, Diogo BG Santos, Raimundo M Nascimento, George LLM Coelho, José C Nicolau, José G Mill, José E Krieger, Alexandre C Pereira

**Affiliations:** 1 Laboratory of Genetics and Molecular Cardiology, Heart Institute (InCor), University of Sao Paulo Medical School, SP, Brazil; 2 Departament of Medicine, Ouro Preto Federal University, MG, Brazil; 3 Acute Coronary Care Unit, Heart Institute (InCor), University of Sao Paulo Medical School, SP, Brazil; 4 Department of Physiology, Espirito Santo Federal University, ES, Brazil

## Abstract

**Background:**

Recent studies have reported the clinical importance of *CYP2C19 *and *ABCB1 *polymorphisms in an individualized approach to clopidogrel treatment. The aims of this study were to evaluate the frequencies of *CYP2C19 *and *ABCB1 *polymorphisms and to identify the clopidogrel-predicted metabolic phenotypes according to ethnic groups in a sample of individuals representative of a highly admixtured population.

**Methods:**

One hundred and eighty-three Amerindians and 1,029 subjects of the general population of 4 regions of the country were included. Genotypes for the *ABCB1*c.C3435T (rs1045642), CYP2C19*2 (rs4244285), CYP2C19*3 (rs4986893), CYP2C19*4 (rs28399504), CYP2C19*5 (rs56337013), and CYP2C19*17 (rs12248560) polymorphisms were detected by polymerase chain reaction followed by high resolution melting analysis. The CYP2C19*3, CYP2C19*4 and CYP2C19*5 variants were genotyped in a subsample of subjects (300 samples randomly selected).

**Results:**

The CYP2C19*3 and CYP2C19*5 variant alleles were not detected and the CYP2C19*4 variant allele presented a frequency of 0.3%. The allelic frequencies for the *ABCB1*c.C3435T, CYP2C19*2 and CYP2C19*17 polymorphisms were differently distributed according to ethnicity: Amerindian (51.4%, 10.4%, 15.8%); Caucasian descent (43.2%, 16.9%, 18.0%); Mulatto (35.9%, 16.5%, 21.3%); and African descent (32.8%, 20.2%, 26.3%) individuals, respectively. As a result, self-referred ethnicity was able to predict significantly different clopidogrel-predicted metabolic phenotypes prevalence even for a highly admixtured population.

**Conclusion:**

Our findings indicate the existence of inter-ethnic differences in the *ABCB1 *and *CYP2C19 *variant allele frequencies in the Brazilian general population plus Amerindians. This information could help in stratifying individuals from this population regarding clopidogrel-predicted metabolic phenotypes and design more cost-effective programs towards individualization of clopidogrel therapy.

## Background

Clopidogrel, a prodrug, is a thienopyridine that inhibits adenosine diphosphate-induced (ADP) platelet aggregation. It has been prescribed mainly in patients with acute coronary syndromes (with or without ST-segment elevation) and patients submitted to percutaneous coronary intervention. It is a pro-drug that requires hepatic metabolization and activation by the cytochrome P450 (CYP) in order to produce its active metabolite. Metabolization of the drug is achieved by a number of different CYP isoenzymes. Two oxidative steps are essential, which culminates in the active metabolite. In particular, the isoenzyme CYP2C19 is involved in the two steps contributing to an estimated 45% of first step and 21% of its change to the active metabolite [1-3].

Several studies have demonstrated an association between *CYP2C19 *gene polymorphisms and the enzyme activity [4-9]. The most common genetic variation, designated CYP2C19*2 (c.G681A), leads to a splicing defect that functionally affects the enzyme. Other alterations have also been reported as loss-of-function: CYP2C19*3 (c.G636A; stop codon), CYP2C19*4 (c.A1G; transition in the initiation codon), and CYP2C19*5 (c.C1297T; amino acid substitution) [7-9].

CYP2C19*2 allelic variant has been associated with higher levels of ADP-induced platelet aggregation values in clopidogrel-treated patients and, consequently, a higher risk of adverse cardiovascular events, such as the occurrence of stent thrombosis [4-8]. In contrast, an allelic variant recently reported, CYP2C19*17 (c.C806T; 5'-flanking region of the gene), has been associated with increased enzyme function [10]. Thus, individuals harboring this genetic variant could have an enhanced response to antiplatelet treatment with clopidogrel, improving the prevention of thrombotic events, but, on the other hand, having a putative increased risk of bleeding [7,10].

On the other hand, it is known that the bioavailability of clopidogrel and of other drugs is also limited by absorption. The multidrug resistance gene (*MDR1 *or *ABCB1*) encodes a drug-efflux transporter called P-glycoprotein, which functions as a physiologic intestinal barrier against drug absorption [11]. Some studies evaluating patients receiving clopidogrel have reported that the rate of cardiovascular events and the plasma concentrations of clopidogrel active metabolite were different according to the genotype for the polymorphism c.C3435T in the *ABCB1 *gene [8,11].

In addition, it has been observed that the genotypic frequencies of *CYP2C19 *and *ABCB1 *polymorphisms present some degree of variation among different ethnic groups [12,13]. As a result, the prevalence of the predicted metabolic phenotypes associated with these polymorphisms may vary significantly among different world-wide populations, leading the impact of pharmacogenetic testing for these variants extremely dependent on a particular country/population genetic architecture.

The Brazilian population is one of the most heterogeneous in the world, being a mixture of different ethnic groups, composed mainly of European descent, African descent and Amerindians [14]. The main aims of this study were to evaluate the frequencies of *CYP2C19 *and *ABCB1 *polymorphisms and to identify the clopidogrel-predicted metabolic phenotypes according to ethnic groups.

## Methods

### Study Population

This study included 1,029 subjects of the general population selected using the Hearts of Brazil Project (HBP) [15]. The universe of the HBP consisted in the set of inhabitants of Brazilian urban centers with more than 100,000 inhabitants. The HBP sample plan was calculated as 2,500 interviews, distributed in the 5 regions of the country proportionally to the number of inhabitants, per sex and age range, based on data from IBGE (Brazilian Institute of Geography and Statistic). A total of 72 cities were chosen in the five regions. The minimum sample size was set at 15, for smaller towns, up to 400, for the city of Sao Paulo (Additional file 1, Figure S1). In the selected cities, the "households" constituted the second-stage units, with one interview per household. Subjects were separated in self-identified sub-groups according to ethnicity, as Caucasian descent, African descent, or Mulattos (considered racially mixed subjects) [15].

In addition, one hundred and eighty-three Amerindians derived from two different groups (Guarani and Tupinikin; Aracruz Indian Reserve, Espirito Santo State in the Southeast Brazilian coast) were also analyzed.

The study protocol was approved by the involved Institutional Ethics Committees and National Ethic Committee for Human Research (CONEP Register Number 4599), and written informed consent was obtained from all participants prior to entering the study.

### Genotyping

Genomic DNA was extracted from peripheral blood leukocytes following a standard salting-out method [16]. Genotypes for the *ABCB1*c.C3435T (rs1045642), CYP2C19*2 (c.G681A; rs4244285), CYP2C19*3 (c.G636A; rs4986893), CYP2C19*4 (c.A1G; rs28399504), CYP2C19*5 (c.C1297T; rs56337013), and CYP2C19*17 (c.C806T; rs12248560) polymorphisms were detected by polymerase chain reaction (PCR) followed by high resolution melting analysis [17,18] (HRM; Rotor Gene 6000^®^, Qiagen, Courtaboeuf, France) using the primer sequences listed in Table 1.

**Table 1:** Primers used for amplification of the *ABCB1 *and *CYP2C19 *polymorphisms

Name	Primer sequence	Fragment size (bp)	Anelling temperature (°C)
***ABCB1*c.C3435T F**	5' GGGTGGTGTCACAGGAAGAG3'	74	59.6
***ABCB1*c.C3435T R**	5' CCTTCATCGAGTCACTGCCT 3'		
CYP2C19*2 F	5' TGCAATAATTTTCCCACTATCA 3'	83	50.5
CYP2C19*2 R	5' CCTTGCTTTTATGGAAAGTGA 3'		
CYP2C19*3 F	5' CCTTGCTTTTATGGAAAGTGA 3'	75	50.5
CYP2C19*3 R	5' TTTTTGCTTCCTGAGAAACCA 3'		
CYP2C19*4 F	5' GCAAGCTCACGGTTGTCTTA 3'	70	63.4
CYP2C19*4 R	5' TGTGGTCCTTGTGCTCTGTC 3'		
CYP2C19*5 F	5' TGGAAGTTGTTTTGTTTTGCT 3'	110	59.6
CYP2C19*5 R	5' TAAGAGATAATGCGCCACGA 3'		
CYP2C19*17 F	5' AAATTTGTGTCTTCTGTTCTCAAA 3'	100	56.7
CYP2C19*17 R	5' AATCCCAGTTCTGCCAGCTA 3'		

The PCR was performed with addition of fluorescent DNA-intercalating SYTO9^® ^(Invitrogen, Carlsbad, USA). In the HRM phase, the Rotor Gene 6000^® ^measured the fluorescence in each 0.1°C temperature increase in the range of 70-94°C. Melting curve was generated by the decrease in fluorescence with the increase in the temperature; and in analysis, nucleotide changes results in different curve patterns. Samples of the three observed curves were sequenced (ABI Terminator Sequencing Kit^® ^and ABI 377 Sequencer^® ^- Applied Biosystems, Foster City, CA, USA) to confirm the genotypes indicated by HRM.

### Predicted metabolic phenotypes

Regarding the predicted metabolic phenotypes related to *CYP2C19 *polymorphisms, individuals were grouped into distinct phenotypes: extensive metabolizer (EM: wild-type for the *CYP2C19 *polymorphisms), intermediate metabolizer (IM: heterozygous genotype for the loss-of-function *CYP2C19 *polymorphisms and wild-type for the CYP2C19*17), poor metabolizer (PM: homozygous or compound heterozygous genotypes for the loss-of-function *CYP2C19 *polymorphisms) and ultra-rapid metabolizer (UM: heterozygous or homozygous genotypes for the CYP2C19*17 and wild-type for the CYP2C19*2). The predicted metabolic phenotype of individual carrying homozygous or heterozygous genotypes for the CYP2C19*17 polymorphism plus heterozygous genotype for the CYP2C19*2 polymorphism are unknown. The individuals carrying homozygous genotype for the *2 (*2/*2) plus heterozygous or homozygous genotype for the *17 were classified as PM, because the first alteration leads to a loss-function enzyme, while a second causes an increased transcription (Additional file 1, Table S1) [6,7].

### Statistical Analysis

Chi-square test was performed for comparative analysis of the allelic and genotypic frequencies for the *ABCB1 *and *CYP2C19 *polymorphisms and of the predicted metabolic phenotype frequency according to ethnicity (Amerindian, Caucasian descent, Mulatto, African descent). Chi-square test was also performed for the determination of differences of allelic frequency for the *ABCB1 *and *CYP2C19 *polymorphisms, of ethnicity according to Brazilian regions, and for the comparative analysis between our data and previous reports. Hardy-Weinberg equilibrium and linkage disequilibrium analyses were conducted with Haploview 4.0. All other statistical analyses were carried out using the SPSS software (v. 16.0), with the level of significance set at p < 0.05.

## Results

### General data of the studied sample

Of the 1,212 subjects (mean age 43.2 ± 14.2), 651 (53.7%) were female and 561 (46.3%) male. Ethnic group distribution was: Amerindians 15.1% (n = 183), Caucasian descent 50.7% (n = 615), Mulatto 26.0% (n = 315) and African descent 8.2% (n = 99).

Of the 1,029 subjects from the general population, 615 (59.8%) individuals belonged to the Southeast region, 198 (19.2%) to the Northeast region, 114 (11.1%) to the South region, 98 (9.5%) to the Midwest region, and only 4 (0.4%) to the North region of the country. The South region presented the highest frequency of Caucasian descent subjects (86.0%) as opposed to the Northeast region (52.0%), while the Northeast and Midwest regions presented higher frequencies of African descent subjects (11.1% and 9.2%) as opposed to the South region (4.4%) (p < 0.001). These results are in accordance to the described by the IBGE (Brazilian Institute of Geography and Statistic) on the last Brazilian census report.

### Frequencies of the *ABCB1 *c.C3435T polymorphism

The frequencies of the variant allele (51.4%) and of the homozygous genotype (27.3%) for the *ABCB1*c.C3435T polymorphism were higher in Amerindians compared with Caucasian descent (43.2%; 19.8%), Mulatto (35.9%; 13.3%), and African descent (32.8%; 13.1%), respectively (p < 0.001) (Table 2).

**Table 2:** Allelic and genotypic frequencies of *ABCB1 *and *CYP2C19 *gene polymorphisms according to ethnic groups

Nucleotide Change and Genotype n (100%)	Amerindian 183	Caucasian descent 615	Mulatto 315	African descent 99	*p ***value**
***ABCB1 *c.C3435T**					
CC	45 (24.6%)	206 (33.5%)	131 (41.6%)	47 (47.5%)	
CT	88 (48.1%)	287 (46.7%)	142 (45.1%)	39 (39.4%)	<0.001
TT	50 (27.3%)	122 (19.8%)	42 (13.3%)	13 (13.1%)	
T allele	51.4%	43.2%	35.9%	32.8%	<0.001
CYP2C19*2, c.G681A					
GG	151 (83.1%)	439 (71.4%)	222 (70.5%)	65 (65.7%)	
GA	24 (13.1%)	144 (23.4%)	82 (26.0%)	28 (28.3%)	0.013
AA	7 (3.8%)	32 (5.2%)	11 (3.5%)	6 (6.0%)	
A allele	10.4%	16.9%	16.5%	20.2%	0.007
CYP2C19*17, c.C806T					
CC	134 (73.2%)	425 (69.1%)	201 (63.8%)	55 (55.6%)	
CT	40 (21.9%)	158 (25.7%)	94 (29.8%)	36 (36.4%)	0.065
TT	9 (4.9%)	32 (5.2%)	20 (6.4%)	8 (8.0%)	
T allele	15.8%	18.0%	21.3%	26.3%	0.048

*ABCB1*c.C3435T (rs1045642); CYP2C19*2 c.G681A (rs4244285); CYP2C19*17 c.C806T (rs12248560)*. p *values were calculated using Chi-square test.

### Frequencies of the *CYP2C19 *polymorphisms

The frequencies of the CYP2C19*2 variant allele and of the homozygous genotype were higher in African descent individuals (20.2% and 6.0%) and were lower in Amerindians (10.4% and 3.8%) compared with other ethnic groups (p = 0.013 and p = 0.007, respectively) (Table 2).

The CYP2C19*3 and CYP2C19*5 variant alleles were not detected in a subsample of subjects from this study (300 samples randomly selected). However, the CYP2C19*4 variant allele was observed in two samples in the heterozygous form (allelic frequency of 0.3%).

The frequency of the CYP2C19*17 variant allele was higher in African descent subjects (26.3%) and was lower in Amerindians (15.8%) compared with other ethnic groups (p = 0.048) (Table 2).

### Linkage disequilibrium analysis between CYP2C19*2 and CYP2C19*17 variant alleles according to ethnic groups

Linkage disequilibrium analysis show that the CYP2C19*2 and CYP2C19*17 variant alleles are in different linkage disequilibrium patterns depending on ethnic origin (Amerindian: 12; Caucasian descent: 54; Mulatto: 53; African descent: 1).

### Frequencies of the *ABCB1 *and *CYP2C19 *variant alleles according to Brazilian regions

Higher *ABCB1*c.C3435T variant allele frequency was observed in the South region (44.0%), while a lower prevalence was presented in the Midwest region (32.0%) (p = 0.017). The Midwest region presented higher frequency of subjects carrying CYP2C19*2 variant allele (26.0%) compared with other regions (p = 0.003). For the CYP2C19*17 variant allele no difference in the frequency between regions was observed (Additional file 1, Figure S2).

### Frequencies of the predicted metabolic phenotypes according to ethnic groups

The proportion of EM individuals was significantly higher in Amerindians (62.3%) than in Caucasian descent (47.0%), Mulatto (40.6%) and African descent (33.3%) (p < 0.001). The frequency of IM individuals was higher in African descent subjects (19.2%) than in other groups (p = 0.010). African descent subjects (32.3%) presented a higher frequency of the UM predicted phenotype, while the Amerindians presented lower frequency (20.8%) (p = 0.048). However, no differences were observed in the frequency of PM individuals according to ethnicity (Table 3) (p = 0.549) (Additional file 1, Table S1 and Table S2).

**Table 3:** Distribution of predicted metabolic phenotypes and observed genotypes according to ethnic groups

Gene n (100%)	Predicted phenotype	Observed genotypes	Amerindian 183	Caucasian descent 615	Mulatto 315	African descent 99	*p ***value**
	**UM**	*17/*17, *1/*17	38 (20.8%)	150 (24.4%)	94 (29.8%)	32 (32.3%)	0.048
	**EM**	*1/*1	114 (62.3%)	289 (47.0%)	128 (40.6%)	33 (33.3%)	<0.001
***CYP2C19***	**IM**	*1/*2	16 (8.8%)	110 (17.9%)	63 (20.1%)	19 (19.2%)	0.010
	**PM**	*2/*2	7 (3.7%)	32 (5.2%)	11 (3.5%)	6 (6.1%)	0.549
	**Unknown**^**a**^	*2/*17	8 (4.4%)	34 (5.5%)	19 (6.0%)	9 (9.1%)	0.429

CYP2C19*2 c.G681A (rs4244285); CYP2C19*17 c.C806T (rs12248560). UM: ultra-rapid metabolizer; EM: extensive metabolizer; IM: intermediate metabolizer; PM: poor metabolizer. ^a ^The predicted metabolic phenotypes of the *2/*17 genotypic combination is unknown. *p *values were calculated using Chi-square test.

The predicted metabolic phenotype of individual carrying homozygous or heterozygous genotypes for the CYP2C19*17 polymorphism plus a heterozygous genotype for the CYP2C19*2 polymorphism (*2/*17) are unknown. The frequencies of these genotype combinations were similar in Amerindians (4.4%), Caucasian descent (5.5%), Mulatto (6.0%), and African descent (9.1%) groups (p = 0.429) (Table 3).

## Discussion

Probably the main contribution of the present study was the demonstration, in the Brazilian population of significant differences between the allelic and genotypic frequencies of polymorphisms associated to clopidogrel, according to ethnicity. To our knowledge this is the first study evaluating *CYP2C19 *polymorphisms in Brazilian Amerindians plus a sample representative of the Brazilian general population. These results could be very useful in the strategic planning of the implementation of pharmacogenetic testing for these variants, aiming at an individual therapeutic approach and adverse drug effect profile prediction both, at the individual and regional country level. To our knowledge, there is no implemented genetic-based prescription program in Brazil; however, the study by Gladding et al concluded that genotyping for the relevant gene polymorphisms may help to individualize and optimize clopidogrel treatment [19].

One potential limitation relates to the self-referred ethnicity. However, this type of classification is the one most probable to be encountered in real-life situations. In addition, even self-referred ethnicity was able to clearly differentiate groups of individuals with rather different allele and genotype frequencies. Finally, different allele and genotype frequencies were also observed when individuals were separated by the country's geographic region. Although a great amount of this effect was probably due to different ethnic distribution according to regions, both variables were independently predictive of genotype distribution in multivariate models adjusted by both ethnic group and country region (data not shown). Probably interaction effects, as well as other confounders, could explain these findings. In fact, one should not dissociate ethnic group self-referral from country region, since cultural aspects may modulate the way people describe themselves regarding ethnicity and this certainly may differ in different parts of the country. This effect should be studied in future works.

Similarly, interethnic differences were observed in the *VKORC1 *polymorphism in 4,886 individuals of 11 countries (Asians, African descent and Caucasian descent), and the contribution of *VKORC1 *toward dose requirements is higher in whites than in nonwhites [20]. Other study reported the worldwide allele frequency distribution of four genetic polymorphisms known to influence warfarin dosing and concluded that understanding the worldwide distribution of markers is important for the future application of pharmacogenomic-based algorithms to different population groups [21].

The *ABCB1 *gene encodes a P-glycoprotein that exports drugs, including clopidogrel. Simon et al (2009) studied 2,208 patients presenting with an acute myocardial infarction and receiving clopidogrel therapy. They reported that patients carrying one or two variant alleles for the *ABCB1 *c.C3435T polymorphism presented more than five times the rate of adverse events of patients with the wild-type genotype [8].

The *ABCB1 *c.C3435T variant allele frequencies reported in studies using samples from African individuals (Ghanian and Kenyan [22]) was of 17.0%. We observed in African descent subjects a higher frequency (32.8%) than in the two cited studies, but this frequency was lower than in other ethnic groups of our study (p < 0.001). In studies of Caucasians subjects (French, 43.0% [23]; Spanish, 48.0% [24]; German, 48.0% [25]; Polish, 49.0% [26]), the variant allele frequency was similar (p > 0.050) to the observed in our sample of Caucasian descent (43.2%) and Amerindian (51.4%) individuals. This result is probably explained by undetected (or unreferred) admixture history of the studied individuals self-referred as African descent from the Brazilian population.

The absence of CYP2C19*3 and CYP2C19*5 variant alleles and the rare frequency of CYP2C19*4 variant allele (0.3%) in our study does not exclude the existence of individuals with PM or IM predicted metabolic phenotypes by presence of these loss-of-function alleles in the Brazilian population. Nevertheless, it clearly shows that these variants are less powerful for the cost-effectiveness design of programs aiming at the identification of individuals harboring predicted metabolic phenotypes compared to the CYP2C19*2 and *17 alleles.

The CYP2C19*2 variant allele frequency found in our study was higher (p < 0.05) in African descent subjects (20.2%) compared with other ethnic groups and with Italian [27] (11.1%); European American [13] (13.0%); and German [28] (15.9%) subjects. However, this frequency was similar (p > 0.05) to the observed in samples of other African descent populations: African American [13] (25.0%), and African Venda [29] (22.0%).

In the general population, the PM predicted metabolic phenotype frequency (4.8%; 49/1029) was lower (p < 0.050) than in Japanese [30] (15.0%). In contrast, it was higher (p < 0.05) than in European American [13] (2.0%) and in Italians [27] (1.7%).

The phenotypic implications of the CYP2C19*2 polymorphism have been established in recent studies using clopidogrel. Mega et al (2009) observed that carriers of a reduced-function CYP2C19*2 allele have significantly lower levels of the active metabolite of clopidogrel, diminished platelet inhibition, and a higher rate of major adverse cardiovascular events, including stent thrombosis [6]. Simon et al (2009) reported that this genetic variant was associated with an increase in the risk of death, myocardial infarction, or stroke, especially among patients undergoing percutaneous coronary intervention [8].

The CYP2C19*17 variant allele, and the UM predicted phenotype, prevalence found was higher in African descent subjects (26.3%; 32.3%) and lower in Amerindians (15.8%; 20.8%) compared with other ethnic groups (p = 0.048). Some studies have reported lower frequencies of this variant allele in Chinese (4.0%) [10], Japanese (1.3%) [31] and Korean (0.3%) [32]; and similar frequencies in Swedish (20.0%) [32]; and Greek (19.6%) [33]. CYP2C19*17 is a recently reported variant causing ultra-rapid metabolism of CYP2C19 substrates. Regarding clopidogrel, Sibbing et al. (2010) observed that patients carrying heterozygous or homozygous genotypes (UM predicted phenotype) had lower ADP-induced platelet aggregation values compared with wild-type. In addition, CYP2C19*17 allele carriage was significantly associated with an increased risk of bleeding [7].

It is interesting that the genetic and functional knowledge of a CYP2C19 enzyme provide enough information to identify patients who will fall outside the therapeutic window of clopidogrel. Thus, it would be possible to justify increases in dosage or a change in therapy. However, it is important to consider that this metabolism has many influences (other genetic markers, concomitant disease processes, medications, foods, age and lifestyle), all of which culminate in variations in the drug action and in the effectiveness of the therapeutic regimen [9,34].

## Conclusion

Our findings indicate the existence of interethnic differences in the *ABCB1 *and *CYP2C19 *variant allele and predicted metabolic phenotype frequencies in the Brazilian general population plus Amerindians. Recent studies, have reported the clinical importance of these polymorphisms. The results of the present study will be very useful in the development of effective programs in stratifying individuals regarding clopidogrel-predicted metabolic phenotypes.

## Competing interests

The authors declare that they have no potential conflicts of interest regarding the present publication

## Authors' contributions

PCJLS carried out the molecular genetic studies, statistical analysis and drafted the manuscript. RAGS and DGBS carried out the molecular genetic studies. ACP participated in the design of the study, statistical analysis and coordinated experiments and manuscript preparation. RMN, GLLMC, JCN, JGM, JEK participated in the design of the study and were responsible for individual selection and characterization. All authors contributed critically to the manuscript, whose present version was read and approved by all.

## Pre-publication history

The pre-publication history for this paper can be accessed here:

http://www.biomedcentral.com/1471-2350/12/13/prepub

## Supplementary Material

Additional file 1 Table S1. Classification of predict metabolic phenotype according to genotype combinations for the CYP2C19*2 and CYP2C19*17 polymorphisms. Classification in EM, IM, PM, UM. Table S2. Distribution of genotype combinations for the CYP2C19*2 and CYP2C19*17 polymorphisms according to ethnic groups. CYP2C19*2 genotypes versus CYP2C19*17 genotypes. **Figure S1. Map of Brazil according geographic regions**. Studied cities. **Figure S2. Map of Brazil according geographic regions showing distribution of variant allele frequencies**. Distribution of CYP2C19*2, CYP2C19*17 and *ABCB1 *polymorphisms. Click here for file
