# Supplementary material for: CYP2C19 and ABCB1 gene polymorphisms are differently distributed according to ethnicity in the Brazilian general population
Source: BMC Med Genet. 2011 Jan 19;12:13. doi: 10.1186/1471-2350-12-13 (PMC3033793; doi:10.1186/1471-2350-12-13)
Supplement: Additional file 1 — Table S1. Classification of predict metabolic phenotype according to genotype combinations for the CYP2C19*2 and CYP2C19*17 polymorphisms. Classification in EM, IM, PM, UM. Table S2. Distribution of genotype combinations for the CYP2C19*2 and CYP2C19*17 polymorphisms according to ethnic groups. CYP2C19*2 genotypes versus CYP2C19*17 genotypes. Figure S1. Map of Brazil according geographic regions. Studied cities. Figure S2. Map of Brazil according geographic regions showing distribution of variant allele frequencies. Distribution of CYP2C19*2, CYP2C19*17 and ABCB1 polymorphisms. [file 1471-2350-12-13-S1.DOC]

# Additional files

Table 1. Classification of predicted metabolic phenotype according to genotype combinations for the *CYP2C19*2* and *CYP2C19*17* polymorphisms

|  |  | ***CYP2C19*2*** | | |
| --- | --- | --- | --- | --- |
|  |  | ***1/*1** | ***1/*2** | ***2/*2** |
| ***CYP2C19*17*** | ***1/*1** | **EM** | **IM** | **PM** |
|  | ***1/*17** | **UM** | **Unknown** | **PM** |
|  | ***17/*17** | **UM** | **Unknown** | **PM** |

*CYP2C19*2* c.G681A (rs4244285); *CYP2C19*17* c.C806T (rs12248560)*.* The predicted metabolic phenotype of individual carrying *1/*17 or *17/*17 genotypes for the *CYP2C19*17* polymorphism plus *1/*2 genotype for the *CYP2C19*2* polymorphism are unknown.

Table 2. Distribution of genotype combinations for the *CYP2C19*2* and *CYP2C19*17* polymorphisms according to ethnic groups

|  | ***CYP2C19*2* genotype** | | | |  |
| --- | --- | --- | --- | --- | --- |
|  | ***1/*1** | ***1/*2** | ***2/*2** | | |
| ***CYP2C19*17* genotype** |  |  | |  |  |
|  | **Amerindian** | | | |  |
| ***1/*1** | 114 (62.3%) | 16 (8.8%) | | 4 (2.2%) |  |
| ***1/*17 or *17/*17** | 38 (20.8%) | 8 (4.4%) | | 3 (1.5%) |  |
|  | **Caucasian descent** | | | |  |
| ***1/*1** | 289 (47.0%) | 110 (17.9%) | | 26 (4.2%) |  |
| ***1/*17 or *17/*17** | 150 (24.4%) | 34 (5.5%) | | 6 (1.0%) |  |
|  | **Mulatto** | | | |  |
| ***1/*1** | 128 (40.6%) | 63 (20.1%) | | 10 (3.2%) |  |
| ***1/*17 or *17/*17** | 94 (29.8%) | 19 (6.0%) | | 1 (0.3%) |  |
|  | **African descent** | | | |  |
| ***1/*1** | 33 (33.3%) | 19 (19.3%) | | 3 (3.0%) |  |
| ***1/*17 or *17/*17** | 32 (32.3%) | 9 (9.1%) | | 3 (3.0%) |  |

*CYP2C19*2* c.G681A (rs4244285); *CYP2C19*17* c.C806T (rs12248560)*.* The predicted metabolic phenotype of individual carrying *1/*17 or *17/*17 genotypes for the *CYP2C19*17* polymorphism plus *1/*2 genotype for the *CYP2C19*2* polymorphism are unknown.

**Figures**
